# Study of Prevalence and Response to Needle Stick Injuries among Health Care Workers in a Tertiary Care Hospital in Delhi, India

**DOI:** 10.4103/0970-0218.62565

**Published:** 2010-01

**Authors:** Rahul Sharma, SK Rasania, Anita Verma, Saudan Singh

**Affiliations:** Department of Community Medicine, VMMC & Safdarjung Hospital, New Delhi, India

**Keywords:** Needle stick injury, health care workers, HIV

## Abstract

**Background:**

Because of the environment in which they work, many health care workers are at an increased risk of accidental needle stick injuries (NSI).

**Objective:**

To study prevalence and response to needle stick injuries among health care workers.

**Materials and Methods:**

Study Design: Cross-sectional study. Setting: A tertiary care hospital in Delhi. Participants: 322 resident doctors, interns, nursing staff, nursing students, and technicians. Statistical Analysis: Proportions and Chi-square test.

**Results:**

A large percentage (79.5%) of HCWs reported having had one or more NSIs in their career. The average number of NSIs ever was found to be 3.85 per HCW (range 0-20). 72 (22.4%) reported having received a NSI within the last month. More than half (50.4%) ascribed fatigue as a cause in their injury. Most of the injuries (34.0%) occurred during recapping. In response to their most recent NSI, 60.9% washed the site of injury with water and soap while 38 (14.8%) did nothing. Only 20 (7.8%) of the HCWs took post-exposure prophylaxis (PEP) against HIV/AIDS after their injury.

**Conclusions:**

The occurrence of NSI was found to be quite common. Avoidable practices like recapping of needles were contributing to the injuries. Prevention of NSI is an integral part of prevention programs in the work place, and training of HCWs regarding safety practices indispensably needs to be an ongoing activity at a hospital.

## Introduction

Because of the environment in which they work, many health care workers are at an increased risk of accidental needle stick injuries (NSI). As a result, these workers are at risk of occupational acquisition of blood borne pathogens such as HIV, hepatitis B and C, and other diseases. The average risk of transmission of HIV to a health care worker after percutaneous exposure to HIV-infected blood has been estimated as 3 in 1000.(1,2) According to a WHO study, the annual estimated proportions of health-care workers (HCW) exposed to blood-borne pathogens globally were 2.6% for HCV, 5.9% for HBV, and 0.5% for HIV, corresponding to about 16,000 HCV infections and 66,000 HBV infections in HCW worldwide.(3)

Because needle stick injuries are often under reported, health care institutions should not interpret low reporting rate as low injury rate. Injuries recorded through standard occupational reporting systems may underestimate the true injury rate, as much as 10-fold.(4) Needle stick injuries have significant indirect consequences in health care delivery especially so in the developing countries, where already the qualified work force is limited with respect to the disease burden in the population. These injuries not only potentiate health consequences but also cause emotional distress in health care workers which results in missed workdays and directly affects the health care services and resources.

The present study addresses this important issue of NSI and aims at determining their occurrence among the health care workers in a tertiary care hospital in Delhi, the various factors responsible for needle stick injuries, the circumstances under which they occur and explores the responses of the health care workers after an injury.

## Materials and Methods

This was a cross-sectional study among health care workers on details of needle stick injury. The population under study included senior residents, junior residents, interns, nursing staff and students, and lab technicians working in the various clinical departments of a large tertiary care hospital in Delhi. Permission for carrying out the study was taken in advance. A purposive sampling was done aimed at covering at least 40 respondents among each type of health care workers, working in departments where exposure to needle stick injury may occur. Data collection involved the simple interviewing technique using a semi-open questionnaire that was filled by the interviewer.

The health care workers were contacted in person and told about purpose of the study and that their responses shall be kept anonymous. Informed consent was taken from each respondent before conducting the interview. The inclusion criteria were health care workers in the hospital, both male and female and including those professionals who normally deal with needles- SRs, JRs, interns, nursing staff and students, and lab technicians. Exclusion criteria were all professors, specialists and consultants and the health care workers of the departments who do not normally involve use of needles.

Needle stick injury was defined as “any cut or prick to the respondents by a needle previously used on a patient is work related and sustained within the hospital premises.” Data thus collected were entered into a computer-based spreadsheet for analysis. The statistical tests applied included proportions and Chi-square tests for significance of associations.

## Results

The study was carried out in a large tertiary care hospital in Delhi in the month of March 2008. The respondents included 322 health care workers of the hospital, consisting of 64 senior residents, 47 junior residents, 74 interns, 52 nursing staff, 42 nursing students, and 43 laboratory technicians.

A large percentage (256 or 79.5%) of HCWs reported having had one or more NSIs in their career, maximum among the nursing students (94.2%). The average number of NSIs ever was found to be 3.85 per HCW (±3.29 SD). Among the HCWs who had been working for at least 1 year, the mean number of NSIs was as high as 4.5 (±3.4 SD). A specific question was asked about NSI injuries during the last month of work. Seventy-two (22.4%) of the respondents reported having received a NSI within the last month. The maximum NSI within last 1 month was reported among three groups-senior residents (26.6%), lab technicians (25.6%), and nursing students (25.0%). This is depicted in Table 1.

**Table 1:** Responses of the various categories of the health care workers to different questions regarding their needle stick injury (n = 256 who had ever had a needle stick injury)

	Interns	Junior residents	Senior residents	Nursing students	Staff nurses	Lab technicians	*P* value
Proportion who had a NSI within the last 1 month*	16.2	21.3	26.6	25.0	21.4	25.6	0.73
Proportion who had been wearing gloves at the time of their last NSI	79.2	81.6	71.2	77.6	55.3	67.7	0.096
Proportion who received their injury while recapping the needle	29.2	31.6	36.5	28.6	34.2	48.4	0.048
Proportion who reported their NSI to a supervisor or senior	31.3	35.1	23.1	38.8	23.7	6.5	0.03
Proportion who took PEP after their most recent NSI	6.3	7.9	13.4	6.1	5.2	6.5	0.31

All figures represent % responses for each category of health care worker;

* n = 322 for this question, NCI - Needle stick injuries, PEP - Post-exposure prophylaxis

Among the respondents, 45 (17.6%) reported having ever had a NSI involving a high-risk patient, “high-risk” being defined as known history of HIV, hepatitis B or C, or IV drug use. This was more among the doctors (SRs, JRs and interns) being about 21% in each and least among the lab technicians (9.7%) who are less likely to be knowing the clinical history of the patient. The questions asked thereafter pertained to the most recent NSI that the HCWs had got. Among the 256 respondents who had ever received a NSI, 70 (27.3%) had not been wearing gloves at the time of the incident. Staff nurses (44.7%), lab technicians (32.3%), and senior residents (28.8%) were found to be most likely not to be wearing gloves. Of the respondents, 216 (84.4%) attributed the NSI as having been self-caused, while the remaining 15.6% attributed to someone else. A majority (178 or 69.5%) of the NSI were from a hollow-bore type of needle, with solid-bore needle being involved in only 30.5% incidents of injury. In 161 (62.9%) of the NSI incidents, there was active bleeding from the wound.

Information was also elicited regarding the timing of the injury. In 75 (29.4%) the injury occurred during use of the needle, with the greater part of injuries (167 or 65.5%) occurring after use but before disposal, and 13 (5.1%) during disposal of the needle. The HCWs had been at work continuously for an average 15.8 h (range 02-28 hours) when the most recent injury happened.

A semi-open-ended question was asked as to what the HCWs thought to be the cause of their recent NSI. The results are depicted in Table 2. More than half (50.4%) ascribed fatigue as a cause in their injury. Interestingly, 28 (10.9%) believed that the NSI could not have been prevented. Most of the injuries (34.0%) occurred during recapping. In 75 (29.3%) cases, the NSI occurred while handling the needle, in 54 (21.1%) due to collision with another person, and in 39 (15.2%) due to manipulation by patient.

**Table 2:** Cause of their most recent needle stick injury as per the health care workers who had received a needle stick injury (n = 256)

Cause of the injury	N	%
Lack of assistance	69	27.0
Fatigue	129	50.4
Rushed	30	11.7
Could not have been prevented	28	10.9

The responses to the question “What did you do after the needle stick injury?” are depicted in Table 3. Only 53 (20.7%) of the HCWs got their blood tested immediately after the injury. Thirty-six (14.1%) got their blood test done again after an interval of time. Of the HCWs who received an injury, only 70 (27.5%) reported it to a supervisor or senior.

**Table 3:** Response of the health care workers after the most recent needle stick injury (n = 256)

Response after the injury	N	%
Nothing	38	14.8
Washed with water	22	8.6
Washed with water and soap	102	39.8
Applied spirit	27	10.5
Post-exposure prophylaxis	01	0.3
Washed with water and applied spirit	11	4.3
Washed with water and soap and applied spirit	35	13.7
Washed with water and soap and applied spirit and post-exposure prophylaxis	16	6.3

Note: The total (and %) do not add up to 100% as some infrequent combination of responses are not shown

## Discussion

A large majority (79.5%) of the workers reported having received a NSI in their career, which is a concerning number. A study in rural North India too had found a similar prevalence of NSI ever in working lifetime to be 73%.(5) Several other studies too have consistently found that a very high proportion of HCWs have received needle stick injuries while performing their work, both in India and internationally.(6–11)

In our study there were 93 incidents of NSI among the 322 HCWs within past month, giving an occurrence rate of about 3.47% per annum. Pournaras *et al*. had found the incidence of NSI to be 2.4% per year, but which they themselves discuss to be apparent underreporting as they considered only reported incidents.(11) A large multinational study by WHO on global burden of sharps injury estimated the average number of injuries per HCW to be 0.2-4.7 sharps injuries per year.(3) In our study, the average number of NSIs ever for a HCW was 3.85 (4.5 if we consider the HCWs who had been working at least 1 year), which compares well with finding of 4.2 by Kermode *et al*.(5)

Wearing gloves is known to be an important line of defense but several of the HCWs had not been wearing them at the time of their injury, higher proportions among the nurses and the technicians. Most (84.4%) of the injuries were admitted to be because of error by self, a figure similar to earlier findings.(6) In the present study too, most of the injuries (70%) were from a hollow-bore needle as observed previously too.(8,12) An important finding was that a majority of the injuries occurred not during use itself, but rather during the handling between use and its disposal, as seen earlier too.(8) The training programs regarding dealing with needles and sharps usually jump from precautions during use directly to safety during discarding the needle. During safety training programs, it should be emphasized that there is need to maintain utmost care and caution during the in-between handling also.

The HCWs had been at work continuously an average 15.8 h before their most recent injury. In fact 50.4% of the HCWs who had got a NSI ascribed their injury to fatigue. Long working hours have been found to be an important risk factor for NSI.(13,14) The health care environment in a tertiary care hospital is a hectic and stressful one, and long duty hours are common. It must be ensured that people putting in long hours continuously get to take short breaks in between, to refresh themselves up. Several studies have shown recapping to be an important cause of NSI.(3,5,8,11,15) All training programs emphasize that recapping of needles after use is not to be done. Still in our study too, most of the injuries (34.0%) occurred during recapping. There is need for safety training to be a regular activity with periodic bolstering. IEC material should be displayed prominently at the places of work, emphasizing the point about no recapping.

In present study while 60.9% washed the site of injury with water and soap, a matter of concern is that 14.8% did nothing following their most recent NSI. Only 20 (7.8%) of the HCWs took post-exposure prophylaxis (PEP) against HIV/AIDS after their injury. This included all 11 (4.3%) who knew their NSI to be from a “high risk” patient. Very few of the NSIs get reported to the health care system. In our study too, only about one in four (27.5%) of the HCWs reported their injury to a supervisor or senior. Previous studies too have shown a wide difference in the occurrence rate of NSI in the studies which asked directly from the HCWs compared to those relying only on those who self-reported to the institute.(4)

Needle stick injuries represent an omnipresent occupational hazard that people working in a hospital face daily. While no NSI can be regarded as ‘could not have been prevented’ as nearly 11% of our sample answered, it may not be practically feasible to avoid their occurrence altogether. But undoubtedly, their occurrence can be minimized to a large extent. Prevention of NSI is the best way to prevent several diseases in health care workers. It should be an integral part of prevention programs in the work place, and training of HCWs regarding safety practices indispensably needs to be an ongoing activity at the hospital. It is recommended that every hospital should develop a multi-pronged strategy to deal with NSI. Besides health promotion, there should be setting up of an adequate surveillance mechanism in every large hospital and also of facilities for prompt response and treatment of NSI.
